# Diet, the Gut Microbiome, and Estrogen Physiology: A Review in Menopausal Health and Interventions

**DOI:** 10.3390/nu18071052

**Published:** 2026-03-26

**Authors:** Michelle Jing Sin Lim, Elvina Parlindungan, E’ein See, Ching Hwee Gan, Rachel Yap, Germaine Jia Min Yong

**Affiliations:** Singapore Institute of Food and Biotechnology Innovation (SIFBI), Agency for Science, Technology and Research (A*STAR), 31 Biopolis Way, Nanos, Singapore 138669, Singapore; michelle_lim@a-star.edu.sg (M.J.S.L.); elvina_parlindungan@a-star.edu.sg (E.P.); eein_see@a-star.edu.sg (E.S.);

**Keywords:** estrobolome, gut microbiome, perimenopause, personalized nutrition, synbiotics

## Abstract

Menopause represents a key transitional phase in women’s health, characterized by declining estrogen levels and increased risk for cardiometabolic, musculoskeletal, and urogenital disorders. Beyond its endocrine roots, emerging evidence highlights the gut microbiome as a critical modulator of systemic hormonal balance. This review synthesizes current understanding of the bidirectional relationship between estrogen and the gut microbiome and its implications for women’s health during menopause. Evidence from current studies reveals distinct findings across populations, reflecting the complexity of estrogen regulation in part by the gut microbiome (i.e., estrobolome). While no ideal gut microbial composition has been identified for women across stages of perimenopause, likely due to geographically unique gut microbiome profiles among healthy women, greater microbial diversity has been positively associated with improved estrogen regulation. Conversely, reduced diversity and altered *Firmicutes*/*Bacteroidetes* ratios have been linked to biomarkers of inflammation during perimenopause, which is a key driver across many perimenopausal symptoms. Although hormone replacement therapy remains the primary clinical intervention during perimenopause, we highlight emerging evidence on the adjuvant potential of diet, synbiotics, phytoestrogens, and strain-specific probiotics in modulating the estrogen–gut microbiome axis for improved health span trajectories and better symptom management. Future longitudinal studies integrating diet, gut microbiome profiles and symptom trajectories are essential to clarify these mechanisms across ethnicity and geography. Ultimately, understanding localized diet–microbiome interactions will enable the development of accessible, personalized, and non-hormonal strategies to complement and increase agency in proactive management during the perimenopausal transition.

## 1. Introduction

### 1.1. Overview of Menopause

**Figure 1:**
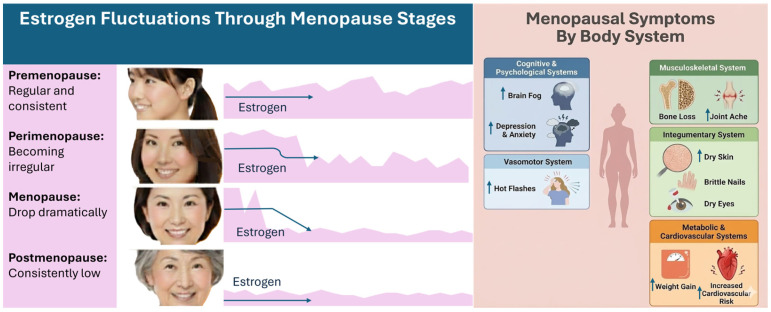
Estrogen fluctuations across menopause stages and common menopausal symptoms stratified by body system [10,11]. Caucasian women have reported a higher prevalence for the following symptoms compared to Asian women (indicated by a blue upward arrow)—brain fog (78.8% vs. 70.4%), depression and anxiety (62.9% vs. 51.9%), hot flashes (65.4% vs. 61.4%), joint ache (65.3% vs. 61.4%), dry skin (61.2% vs. 60.0%), weight gain (80.8% vs. 71.6%), and increased cardiovascular risk (32.1% vs. 30.0%) [12].

Menopause, defined as the absence of menses for twelve consecutive months [1,2], marks the end of a woman’s reproductive years [3]. The preceding phase, perimenopause, can last many years and involves hormonal fluctuations that cause a multitude of symptoms affecting well-being and productivity (Figure 1) [2,4,5,6,7,8]. In the U.S., these symptoms contribute to an estimated $1.8 billion in annual productivity loss [9].

Perimenopause is marked by three key hormonal shifts: erratic fluctuation in estrogen, declining progesterone and elevated follicle stimulating hormone (FSH) [3]. Estrogen production fluctuates with FSH levels and can reach higher concentrations compared to women under age 35 years, while progesterone levels are lower than in mid-reproductive age women and vary inversely with body mass index [13]. Fluctuations and the progressive decline of estrogen and related hormones in women exert profound effects on multiple physiological systems and overall health, and the severity of menopausal symptoms usually varies amongst women, largely influenced by ethnic, regional and socioeconomic factors [14,15,16].

Up to 90% of women will experience some menopausal symptoms, with approximately 40% reporting severe symptoms that reduce their quality of daily life [17,18]. While aging and declining estrogen exhibit overlapping clinical features [19], menopause itself has been associated with more than 30 distinct symptoms [20]. These include symptoms such as weight gain, skin dryness, hair thinning and various cognitive (brain fog, forgetfulness) and psychological indications (low mood, anxiety, depression) [19,21,22]. In addition, urogenital symptoms, such as vaginal dryness, irritation and recurrent urinary tract infections, are also associated with menopause [23]. Symptom patterns and severity can be affected by ethnicity, as Asian women tend to report less concern over vasomotor symptoms (VMS) and more cognitive issues compared to their Caucasian counterparts [24,25,26,27,28,29,30,31,32,33,34]. A large remote cross-sectional study (*n* = 68,864) also showed that symptoms such as brain fog, depression and anxiety, hot flashes, joint ache, dry skin, weight gain, and increased cardiovascular risk were also higher in Caucasian women compared to Asian women (Figure 1) [12]. A recent study on 1741 perimenopausal women across five major Asia Pacific countries reported an overall high symptom burden [35]. The study found that fatigue, joint pain, night sweats and difficulty concentrating were particularly common and resulted in significant disruptions to work performance and daily function in nearly half of the surveyed women [35]. In Singapore, arthralgia is the most reported symptom, with 75% of women experiencing varying degrees of muscle and joint pain [36]. Arthralgia was also observed to be associated with other mobility symptoms, such as poor muscle strength, and with vaginal dryness [36]. Other symptoms reported in Asian women in Singapore include depression (11.8%) [37], anxiety (11.0–19.2%) [37,38], hot flashes (32.9–51.7%) [37,38,39] and dry skin (27.7%) [38].

The decline in estrogen and accumulation of abdominal obesity that occurs during perimenopause have been suggested to be implicated in the development of chronic diseases [22,40]. In addition, the onset of menopause significantly influences long-term health outcomes. A 50% and 10% increased risk of cardiovascular disease and of fatal coronary heart disease and all-cause mortality were respectively observed if menopause occurred before the age of 45 years [18]. Furthermore, women who experienced menopause earlier exhibited poorer cardiovascular, cognitive, bone, urogenital and bladder health, largely due to premature loss of estrogen’s protective effects [21]. The US Study of Women’s Health Across the Nation (SWAN) has further shown that, apart from the loss of estrogen, early perimenopausal women with a history of VMS also have lower bone mineral density than those without VMS [41], which results in higher fracture risk. While cognitive symptoms usually improve after menopause, women with severe symptoms or lower socioeconomic status may experience persistent cognitive decline [22].

### 1.2. Emerging Role of Gut Microbiome as a Modulator of Systemic Hormones

The human gut microbiome plays a pivotal role in maintaining host homeostasis through bidirectional interactions with multiple physiological systems [42,43,44,45]. Emerging evidence highlights a complex interplay between the gut microbiota and systemic hormones, including estrogen [46]. Central to this relationship is the estrobolome, a group of gut bacterial genes encoding β-glucuronidase, β-glucosidase and sulfatase enzymes that regulate estrogen metabolism and circulation [46]. Dysbiosis, or an imbalance in the gut microbiome, can, therefore, disrupt estrogen homeostasis and contribute to the development of estrogen-related diseases [47,48,49]. The estrogen–gut microbiome axis is discussed in greater detail in Section 4 of this review.

### 1.3. Purpose and Scope of Review

This review focuses on examining how the gut microbiome and dietary factors influence health span during perimenopause, highlighting the relationship between estrogen and the gut microbiome during menopausal shifts. This review will also address the potential of diet, synbiotics, phytoestrogens, and strain-specific probiotics in modulating the estrogen–gut microbiome axis and their role in improving symptom management and health span during perimenopause.

## 2. Methods

The search strategy for this narrative review was done using major electronic databases including Google Scholar and Scopus, looking at only studies published in English. For microbiome-related studies, only those published between 1 January 2015 and 31 December 2025 were included for relevancy. Clinical studies were prioritized in this review and included wherever possible; otherwise, *in vitro* studies were mentioned to support the narrative. Some of the search terms used included “perimenopause”, ‘menopause”, “estrogen”, “estrobolome”, “gut microbiome”, “women’s health”, “menopausal symptom”, “probiotics”, “prebiotics”, “hormone replacement therapy”, “fecal microbiota transplantation”, “phytoestrogens”, “traditional Chinese medicine”, “Dan Shen”, and “Kudzu root”.

## 3. Estrogen Physiology and Menopause

### 3.1. Importance of Estrogen in Women’s Health

Estrogen plays a critical role in many bodily functions beyond reproduction [50], and declining estrogen levels during menopause can lead to significant health challenges. Estrogen is considered cardioprotective [51] through improvement of lipid profiles, anti-platelet and anti-oxidant effects, and enhancement of endothelial functions, which collectively help reduce the risk of atherosclerosis [52,53]. Estrogen is also linked to weight management—it raises resting metabolic rate, increases energy expenditure, acts on the brain’s hypothalamus and interacts with glucagon-like pepide-1 (GLP-1), insulin and leptin to control hunger and satiety [54,55,56]. Women with low levels of estrogen during menopause are at higher risk of obesity and metabolic disorders such as type 2 diabetes (T2D) due to unregulated appetite control and insulin resistance [54,57]. Furthermore, literature suggests that estrogen plays a crucial role in delaying cardiovascular events by about a decade in women compared to men [58,59,60,61].

Estrogen’s role in maintaining women’s cognitive function and mood has been reported in the literature. Estrogen modulates receptors in the prefrontal cortex and hippocampus and promotes synaptic density and dendritic spine growth, which are crucial for cognitive function and stress resilience [62]. Moreover, estrogen has also been reported to reduce amyloid-beta accumulation, neuroinflammation, oxidative stress and mitochondrial dysfunction and to improve cholinergic and serotonergic signaling tone [62]. These mechanisms confer neuroprotection against aging and early onset of Alzheimer’s disease and dementia [63]. Estrogen also regulates neurotransmitter systems involved in mood and motivation, including serotonin, dopamine and glutamate, with receptor binding linked to improved mood and reduced depression risk [64,65]. It is also reported that estrogen supports brain health by maintaining blood flow, protecting blood vessels and preserving the blood–brain barrier [66]. As such, its fluctuations and decline during perimenopause may increase the risk of vascular issues and contribute to faster cognitive decline. Thus, it is clear estrogen is essential in maintaining health, and its decline during perimenopause can lead to several clinical consequences, such as increased cardiovascular risk, Alzheimer’s disease, obesity and T2D.

### 3.2. Estrogen Synthesis, Metabolism and Function

Estrogen is mainly synthesized from cholesterol, with about 90% secreted by the ovaries and a small amount produced by the adrenal gland and adipose tissues [67]. There are three main forms of estrogen—estrone (E1), estradiol (E2) and estriol (E3), with E2 being the most prevalent and biologically active during the premenopausal period [50,67]. E1, synthesized in adipose tissue from adrenal hormones, plays a more significant role after menopause, while E3 is the weakest estrogen and is produced primarily in large amounts by the placenta during pregnancy. Estrogen regulates many critical metabolic functions through two types of receptors—classical nuclear receptors (ERα and ERβ) and novel cell surface membrane receptors (GPR30 and ER-X) [50]. Estrogen receptors (ERs), especially ERα, are key regulators of appetite, body weight, fat distribution, inflammation, glucose homeostasis, lipolysis/lipogenesis, energy expenditure, reproduction and cognition [68]. The precise tissue-specific dynamics, signaling pathways and context-dependent receptor modulation by these estrogens, especially in perimenopausal and disease states, are still not fully elucidated.

### 3.3. Changes in Estrogen Level During Menopause

During a woman’s reproductive years, the average level of total estrogen is 100–250 pg/mL; however, the concentration of circulating E2 declines to 10 pg/mL after menopause [69], similar to that in men [70]. It is reported that, during perimenopause, E2 levels are markedly elevated in women due to atypical follicular activity, including luteal out-of-phase (LOOP) and lag cycles [3,71]. These cycles are characterized by high E2 and low progesterone levels, often resulting from the development of large, non-ovulatory follicles [71,72]. As ovarian reserves diminish, the feedback regulation involving inhibin B and FSH becomes disrupted, further contributing to hormonal instability [72,73,74]. In late perimenopause stages, when symptoms are often more severe, estrogen levels tend to wane more consistently, especially during anovulatory cycles, leading to sustained low estrogen levels post-menopause and the emergence of classic menopausal symptoms [13,75,76]. These variations in estrogen throughout perimenopause reflect a need for a differentiated approach in regulating estrogen levels.

### 3.4. Factors Affecting Estrogen Level

Beyond aging, other factors such as genetics, environment, lifestyle and diet also seem to influence estrogen levels.

#### 3.4.1. Genetics

Grub et al. showed that ER gene polymorphisms can significantly modulate the effect of E2 fluctuation, as these lead to variations in ER protein structures [77], which then affect the binding affinity to E2 and the activation of downstream signaling pathways [78]. This could partly explain women’s differential response to E2 fluctuations [79].

#### 3.4.2. Environment

It is well established that environmental contaminants, especially endocrine-disrupting chemicals, can be particularly problematic for women, as these chemicals can accelerate reproductive aging and lead to an earlier onset of menopause [80]. In a cohort of 25,957 women aged 18–65 years, the study found an association between perfluorocarbon (PFC) exposure, decreased E2 and early menopause in women over 42 years old, validating the casual role that PFCs play in disrupting proper endocrine disruption [81]. In another study, researchers observed that increases in exposure to air pollutants NO_2_, O_3_ and particulate matter less than 2.5 microns (PM_2.5_) were linked to significant declines in E2 and FSH levels across menopausal stages, suggesting the detrimental impact of air pollutants on women’s reproductive hormones [82].

#### 3.4.3. Lifestyle and Diet

Recent evidence has emerged that lifestyle and diet can also affect estrogen production. Studies have demonstrated that dietary patterns resembling the Mediterranean diet can lower circulating E2 levels and improve estrogen metabolite profiles, resulting in a reduction in weight, blood pressure, blood ω6:ω3 ratio, triglycerides, blood glucose, total cholesterol and low-density lipoprotein (LDL) levels. Such dietary patterns may also improve mood, depression and VMS [83,84]. Weight loss through a combination of diet and moderate-to-vigorous exercise is strongly associated with reductions of 16–20% in systemic E2 levels and increases in sex hormone-binding globulin [85,86], which modulates the bioavailability of estrogen, potentially contributing to lower breast cancer risk [85].

## 4. The Gut Microbiome and Estrogen Metabolism

### 4.1. The Estrobolome Concept

**Figure 2:**
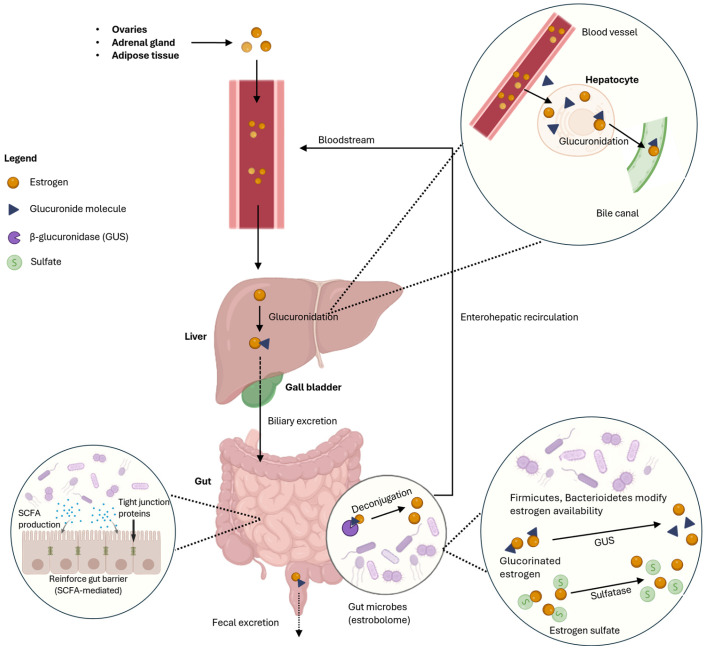
Estrogen—gut microbiome metabolism axis. Diagram made using BioRender.com.

The gut microbiome modulates circulating estrogen levels through enterohepatic recirculation [67]. Estrogens synthesized in the ovaries, adrenal glands and adipose tissues undergo glucuronidation, a conjugation process that facilitates their excretion into the intestinal tract via bile. Once in the gut, bacterial genes collectively known as the estrobolome encode β-glucuronidase, which deconjugates these glucuronidated estrogens and restores their biological activity [47,48]. The reactivated estrogens are subsequently reabsorbed across the intestinal epithelium and returned to systemic circulation for the body to reuse (Figure 2) [87].

The gut microbial β-glucuronidase (GUS) gene, a key component of the estrobolome, has been detected across several bacterial taxa, particularly within the Firmicutes and Bacteroidetes phyla [47,88,89,90]. Shin et al. reported that women with higher circulating E2 levels exhibited a higher *Bacteroidetes* to *Firmicutes* ratio, supporting the role of these taxa in estrogen regulation [91]. Among the microbial enzymes, β-glucuronidase plays a central role by reactivating deconjugated estrogens, hence influencing enterohepatic circulation and systemic estrogen bioavailability [48].

Menopause is associated with reduced gut microbiome diversity [67], and the relevance of the estrobolome appears to vary by life stage. In premenopausal women, fecal microbiome richness shows little correlation with estrogen, whereas in postmenopausal women, gut diversity correlates strongly with circulating non-ovarian estrogen levels [92]. This suggests that microbial modulation of estrogen becomes more pronounced as endogenous estrogen production declines.

### 4.2. Microbial Deconjugation and Enterohepatic Circulation

In premenopausal women, estrogens are predominantly synthesized in the ovaries and placenta, while the kidney, adipose tissue, skin, and brain contribute more significantly after menopause as ovarian estrogen declines [50,93]. With systemic estrogen depletion, receptor-mediated regulation becomes more prominent. Mosconi et al. observed higher estrogen receptor density in the postmenopausal brain, which is interpreted as a compensatory mechanism to preserve estrogen-dependent neural functions [94]. However, symptoms such as mood changes persist, highlighting that receptor upregulation is unable to fully offset hormonal loss and the need to restore estrogen abundance.

The gut microbiota plays a central role in estrogen metabolism by influencing bile acid homeostasis and reactivation of conjugated estrogens. Conjugated estrogens depend on bile-mediated intestinal transport, where β-glucuronidase-producing bacteria deconjugate them, enabling reabsorption and systemic availability [95]. Altered β-glucuronidase abundance has been associated with variations in estrogen bioavailability, though its effect appears context dependent [96]. For example, β-glucosidase activity correlates positively with E2 in individuals with polycystic ovary syndrome (PCOS) but inversely in the control group [97].

Life stages also influence β-glucuronidase activity. In one study of Hispanic women, postmenopausal women exhibited lower β-glucuronidase abundance than premenopausal counterparts [98], whereas another study of multi-ethnic women reported higher levels post-menopause, suggesting a potential paracrine role in estrogen target tissues [99]. These discrepancies likely reflect demographic and physiological variability, including differences in body mass index (BMI), ethnicity, perimenopausal stage, diet and lifestyle habits, among other confounders, highlighting the complexity of microbial regulation in estrogen metabolism. Further investigation is warranted to determine whether and under what conditions (physiological, pathological, etc.) β-glucuronidase directly modulates systemic estrogen in order to better understand it as a biomarker or therapeutic target in estrogen-sensitive disorders.

### 4.3. Microbial Composition, Diversity and Estrogen Regulation

Estrogen metabolism within the gut is largely mediated by β-glucuronidase and/or β-galactosidase enzymes, both of which are produced by diverse bacterial genera, including *Alistipes*, *Bacteroides*, *Bifidobacterium*, *Citrobacter*, *Clostridium*, *Collinsella*, *Dermabacter*, *Escherichia*, *Faecalibacterium*, *Lactobacillus*, *Marvinbryantia*, *Propionibacterium*, *Roseburia*, and *Tannerella* [48,100,101,102,103,104,105].

Although it is still unclear what is the ideal gut microbiome composition for postmenopausal women, higher gut microbiome diversity is consistently associated with improved estrogen regulation. This is partly supported by the finding that antibiotic-treated mice have lower free E2 than those without treatment [106]. In postmenopausal women and men, non-ovarian estrogen levels correlate strongly with *Clostridia* and *Ruminococcaceae* taxa, whereas such associations are not found pre-menopause [104]. Reduced *Firmicutes* abundance during menopause correlates with higher β-glucoronidase activity, suggesting microbial compensation for estrogen recycling [107]. During puberty, when estrogen levels rise significantly and rapidly, it was found that girls displayed greater compositional shift than boys, particularly among *Ruminococcaceae*, *Lachnospiraceae*, *Bacteroidales*, *Paludibacter*, *Macellibacteroides*, and *Barnesiella* [108]. In ovariectomized rats, the *Bacteroidetes* to *Firmicutes* ratio increased [109], while estrogen supplementation in postmenopausal women reversed this shift [110]. These findings collectively highlight a bidirectional regulatory loop between estrogen and the gut microbiome that evolves across life stages.

In women, higher circulating estrogen levels are generally associated with greater gut microbial diversity [91,111,112]. Several taxa have been shown to correlate with E2 levels. For instance, *Slackia* and *Butyricimonas* are negatively correlated with serum E2 in Korean women [91], whereas *Acinetobacter guillouiae*, *Aggregatibacter segnis*, and *Bifidobacterium animalis* are positively correlated with E2 in Chinese women [113]. Similarly, in Chinese perimenopausal women, *Monologues*, *Facalibaterium*, *Dialister*, and *Lachnospiraceae* were positively correlated with E2 concentrations [114]. These associations collectively support the notion that estrogen availability shapes the ecological landscape of the gut microbiota.

As women transition into menopause, declining estrogen levels are accompanied by distinct alterations in the gut microbiome. While findings across studies vary [98,115,116], several have reported a reduction in microbial alpha diversity in postmenopausal women [98,115] (Table 1). Common compositional patterns include a depletion of Firmicutes, particularly *Roseburia* and *Ruminococcus*, and an enrichment of Bacteroidetes, such as *Bacteroides*, *Parabacteroides* and *Prevotella,* resulting in a lower Firmicutes to Bacteroidetes ratio in postmenopausal women [98,115,116]. However, the specific taxa affected appears to vary across populations. In Chinese cohorts, *Roseburia* was depleted, while *Tolumonas* was enriched in postmenopausal women [115]; in Spanish women, *Haemophilus* and *Dorea* were enriched, and *Clostridium neonatale* was depleted in postmenopausal women [116]. Among Hispanic/Latino women, *Sutterella wadsworthensis* was found in greater abundance, whereas *Akkermansia muciniphila* was depleted in postmenopausal individuals [98]. These differences likely reflect the combined influence of ethnicity, diet and lifestyle on microbial ecology.

Even across the spectrum of menopause symptom severity, distinct gut microbial signatures were observed (Table 1). Liu et al. observed that, although overall gut microbiome (both alpha and beta) diversity did not differ between healthy postmenopausal women and those with menopausal syndrome (MPS), specific taxa varied significantly [113]. Women with MPS exhibited higher levels of *Bifidobacterium longum*, *Bifidobacterium adolescentis*, *Eubacterium biforme* and *Bacteroides ovatus* and lower abundances of *Acinetobacter guillouiae*, *Aggregatibacter segnis*, *Bacteroides coprophilus*, *Bifidobacterium animalis*, *Clostridium celatum*, and *Ruminococcus albus* [113]. Similarly, in perimenopausal women with high FSH and low E2 levels, *Faecalibacterium*, *Subdolibranulum*, *Agathobacter*, *unclassified Lachnospiraceae*, and *Roseburia* were depleted, while *Bacteroides*, *Escherichia-Shigella*, *Bifidobacterium*, and *Blautia* were enriched [114]. These findings suggest that the microbiome may reflect both hormonal status and symptomatic variation, even within comparable life stages.

### 4.4. Host Factors Affecting Gut Microbiome and Estrobolome Activity

The gut microbiome, including the estrobolome, is shaped by host factors, creating a bidirectional network between estrogen signaling and the gut microbiota. Emerging evidence indicates that estrogen, acting via their receptors, can remodel gut community structure, while microbial β-glucuronidase and related enzymes reciprocally regulate host estrogen exposure through enterohepatic recycling as described above. Within this framework, host variation in ER genes, adiposity-related estrogen production and lifestyle factors, such as exercise, smoking, and alcohol consumption, act as key modulators that collectively determine an individual’s gut microbiota, estrogen bioavailability and risk profile for hormone-sensitive conditions like menopause.

#### 4.4.1. Genetic Variations in Estrogen Receptors

For example, activation of ERα, but not ERβ, lowers *Firmicutes* to *Bacteroidetes* ratios, mimicking the non-obese gut microbiome profile [117]. As reduced *Firmicutes* has been associated with greater β-glucuronidase activity [107], gene polymorphisms that increase ERα signaling may hence increase β-glucuronidase activity and estrogen reabsorption.

#### 4.4.2. Abdominal-Obesity-Related Estrogen Production

Higher BMI, especially central adiposity, increases peripheral aromatization of androgens to E1 or E2, resulting in higher baseline E2 production [105]. This increased E2 load passes through hepatic conjugation and biliary excretion into the gut lumen, where it becomes substrate for the estrobolome [105,118]. Obesity is associated with gut dysbiosis, whereby higher *Firmicutes* abundance and higher *Firmicutes*/*Bacteroidetes* ratio are observed [119]. Multiple studies reported elevated β-glucuronidase activity in obesity, suggesting increased microbial potential for estrogen reactivation, although specific deconjugating taxa remain uncharacterized [120,121]. The combination of elevated adipose-derived estrogen and an “obese” microbiome that efficiently deconjugates estrogen enhances enterohepatic recycling and maintains a state of relative estrogen excess, amplifying the risk for hormone-driven breast cancer in postmenopausal women [105].

#### 4.4.3. Lifestyle Factors (Exercise, Smoking and Alcohol)

Regular physical activity increases microbial diversity and shifts the gut community away from an obesity-associated configuration, paralleling improvements in metabolic parameters and reductions in chronic low-grade inflammation. Exercise lowers T2D risk and, via ERα activation, restores a normal BMI low *Firmicutes* to *Bacteroidetes* ratio [117]. In addition, aerobic training has also been shown to increase alpha-diversity, in particular beneficial taxa like *Akkermansia muciniphila*, which promotes short-chain fatty acid production (SCFA) and balanced estrogen metabolism [122]. Current literature provides limited direct evidence on the specific impacts of smoking or alcohol on estrobolome enzyme activity (e.g., β-glucuronidase); while these exposures consistently induce gut dysbiosis [123,124,125], their mechanistic effects on estrogen metabolism are largely inferred through indirect associations with microbial shifts and inflammation.

Altogether, studies highlight that the gut microbiome evolves dynamically with hormonal transitions. Menopause in women is typically marked by reduced microbial diversity and a compositional shift towards patterns similar to men [98,116]. While study outcomes differ due to population diversity, geographic variation and methodological factors [126,127], a consistent trend can be observed, where the loss of estrogen influences microbial ecology, and in turn, microbial alterations may contribute to metabolic and symptomatic manifestations of menopause [113,114]. Studies have also shown that host factors play a role in modulating the gut microbiome and estrobolome activity. This interplay offers opportunities and promising avenues for dietary, prebiotic or strain-specific probiotic interventions, aimed at restoring microbial balance, enhancing estrogen deconjugation homeostasis and mitigating menopausal-associated health effects.

## 5. Clinical Consequence of Gut Microbiome–Estrogen Interactions in Menopause

Menopause-associated changes in the gut microbiome have significant clinical implications, influencing inflammation, metabolism, mental health, bone density and urogenital function. The major bacterial phyla, Firmicutes and Bacteroidetes, constitute over 90% of microbes in the human gut [128]. A reduced *Firmicutes*/*Bacteroidetes* ratio, often seen post-menopause, has been associated with inflammatory and metabolic disorders [129]. Firmicutes, such as *Ruminococcus*, generate SCFAs that maintain gut barrier and energy homeostasis [130,131]. Their depletion compromises gut barrier function, promotes inflammation, and has been associated with depression [132,133]. Conversely, *Bacteroides* enrichment may reflect compensatory but sometimes pathogenic adaptations [134], while a higher abundance of *Prevotella*, *Sutterella*, and *Dorea* has been linked to obesity [135].

Symptom-specific associations between microbes and menopause outcomes further support this connection. In postmenopausal women, a higher Kupperman index (KI) and hot flash scores correlated positively with *Blautia obeum*, *Butyricicoccus pullicaecorum* and *Ruminococcus torques* and negatively with *Lactobacillus delbrueckii* and *Clostridium cocleatum* [113]. These findings suggest that gut dysbiosis underlies several menopause-related symptoms through inflammation and altered hormone metabolism.

The microbial shifts during perimenopause are also linked to differences in cardiometabolic parameters. *Clostridium lactatifermentans*, which was in lower abundance in postmenopausal women, was linked to higher high-density lipoprotein (HDL) and lower metabolic syndrome risk, while *Sutterella wadsworthensis*, which was enriched, was associated with higher blood pressure [98,136]. *Akkermansia muciniphila*, a β-glucuronidase-producing bacterium depleted post-menopause, has been inversely linked to metabolic syndrome [98,137,138]. The decline in *Bifidobacterium animalis*, a probiotic with anti-inflammatory and metabolic benefits [139,140,141,142,143,144], may contribute to menopausal metabolic symptoms.

The gut microbiota is also implicated in postmenopausal osteoporosis, primarily via immune and metabolic regulations [115,145,146,147,148,149,150,151]. Estrogen deficiency is linked to enrichment of *Tolumonas*, *Megamonas* and Fusobacterium (taxa associated with pro-inflammation) and depletion of SCFA-producing genera such as *Romboutsia*, *Collinsella* and *Bifidobacterium*, which support bone formation [115,152,153,154,155,156,157,158,159,160]. These microbial shifts may contribute to osteoporosis by increasing osteoclast activity and reducing bone mineral density.

Estrogen decline in menopause also alters the vaginal epithelium and microbiota [161]. *Lactobacillus* depletion after menopause raises vaginal pH and facilitates pathogen colonization, contributing to genitourinary syndrome in menopause (GSM) [162,163]. Several studies have shown the role of oral probiotics in modulating the gut–vaginal axis, restoring microbial balance and improving vaginal health in women [164,165,166]. Probiotic capsules containing *Lactobacillus rhamnosus* GR-1 and *Lactobacillus reuteri* RC-14 (10^9^ colony forming units (CFU) in equal proportion) were able to modulate and shift the vaginal microbiome of pregnant women with bacterial vaginosis (BV) towards those without the infection after 30 days of intervention [165]. In another study, the probiotic strain *Lactobacillus gasseri* CECT 30648 was able to colonize the vaginal tract in 55.9% of healthy women after oral administration at 10^9^ CFU, thus demonstrating potential in supporting vaginal health [166]. Thus, it is likely that menopause-related dysbiosis of gut microbiota affects the vaginal microbiota and results in the symptoms observed with the onset of menopause, of which further studies are required for verification.

## 6. Intervention Options for Menopausal Symptom Management

### 6.1. Hormone Replacement Therapy

Hormonal replacement therapy (HRT) is currently the standard, first-line treatment for managing vasomotor and urogenital symptoms of menopause [167]. HRT counteracts estrogen deficiency by supplying exogenous estrogen, either alone or combined with progestin, and has been clinically proven to alleviate menopausal symptoms [168]. In a study by Leite et al. [169], the gut microbiome profiles of premenopausal women, postmenopausal women on HRT and those without HRT revealed that HRT users exhibited a microbiota composition more similar to premenopausal women. This is characterized by higher *Prevotella* and lower *Escherichia* and *Klebsiella* abundance. In contrast, non-users displayed increased *Proteobacteria*, reduced *Bacteroidetes* and lower overall microbial diversity [169]. Similar findings were observed in women with premature ovarian insufficiency, where HRT partially restored microbial balance [170]. Collectively, these studies suggest that HRT not only alleviates menopausal symptoms but also restores gut microbial diversity and influences estrogen bioavailability.

Nonetheless, HRT carries notable risks. Combined estrogen–progestin regimens modestly raise the incidence of cardiovascular events such as stroke and venous thromboembolism, particularly when initiated after age 60 years or more than ten years post-menopause [171]. Long-term HRT has also been associated with a 10–30% increase in breast cancer risk [172,173,174] and other side effects, such as vaginal bleeding [175]. Consequently, safety concerns surrounding HRT have driven growing interest in non-hormonal and microbiome-targeted interventions.

### 6.2. Probiotics and Prebiotics

#### 6.2.1. Probiotics

Probiotics, according to the Food and Agriculture Organization (FAO) and the World Health Organization (WHO), are defined as live microorganisms that confer health benefits to the host when consumed in adequate amounts [176]. While probiotics generally promote a healthy gut microbiome, specific probiotics can metabolize estrogens via deconjugation reactions to facilitate their reabsorption and influence systemic estrogen availability [103]. A study by Honda et al. used probiotics with β-glucuronidase activity to modulate estrogen levels in peri- and postmenopausal women [104]—amongst 84 strains screened belonging to lactic acid bacteria and *Bifidobacteria*, specific strains of *Levilactobacillus brevis* and *Lacticaseibacillus rhamnosus* demonstrated the capacity to deconjugate estrogens, with *L. brevis* KABP052 exhibiting the strongest β-glucuronidase activity *in vitro*. A preliminary randomized, placebo-controlled trial showed that a probiotic formula containing *L. brevis* KABP052 preserved serum estrogen compared to placebo (starch), with higher E2 and E1 levels after 12 weeks [104].

Probiotics have also demonstrated the ability to ameliorate menopause-associated metabolic disorder in preclinical models. Using an *in vivo* ovariectomized mouse model, *Bifidobacterium longum* 15M1 was shown to counteract menopausal obesity [150]. Moreover, *Lactiplantibacillus plantarum* 30M5 supplementation in combination with soy isoflavone effectively ameliorated lipid metabolism disturbances better than either intervention alone. This combination altered gut microbial composition, increased SCFA production, and enhanced circulating estrogen [150].

Another preclinical study in an ovariectomized mouse model revealed that *Lactobacillus acidophilus* ATCC 4356 supplementation improved trabecular and cortical bone microarchitecture, enhanced bone mineral density and modulated immune responses by altering the Treg–Th17 balance, suppressing osteoclastogenic cytokines (IL-6, IL-7, TNFα, RANKL) and promoting anti-osteoclastogenic factors (IL-10 and IFN-γ) [177]. This demonstrates promising evidence supporting *L. acidophilus* as a potential osteo-protective probiotic for postmenopausal osteoporosis. In humans, supplementation with a synbiotic containing different probiotic strains in early postmenopausal women attenuated femoral and hip bone loss among participants with osteopenia or higher BMI (BMI ≥ 30) [149]. The intervention also modulated gut microbiome functional pathways, indicating gut–skeletal health benefits of synbiotic supplementation in metabolically compromised or osteopenic postmenopausal populations.

Microbes expressing 3β-hydroxysteroid dehydrogenase have been associated with lower serum E2 levels, causing depressive-like behaviors, thereby indicating the gut microbiota as a therapeutic target for mood disorders [178]. Gut microbiome dysbiosis has been associated with a range of neurological and stress-related disorders, whereas probiotic supplementation has shown promise in restoring microbial homeostasis and alleviating depressive symptoms [179]. For instance, administration of *L. rhamnosus* JB-1 increased γ-aminobutryic acid (GABA) and *N*-acetyl aspartate in the brain, directly linking probiotic intervention to neurotransmitter modulation and altered brain activity [180].

Furthermore, probiotics can be a useful adjuvant to minimize associated risks of HRT in postmenopausal women, including for treatment of symptomatic vaginal atrophy. E3, the weakest of the three primary estrogens, is preferred in postmenopausal therapy due to its localized action on urogenital tissues and minimal systemic stimulation, making it a safer alternative for managing menopausal symptoms while minimizing oncogenic risks. Interestingly, probiotic administration may allow for a dose reduction of E3, thereby further enhancing its safety profile—it was reported that low-dose E3 in combination with *L. acidophilus* resulted in transient increases in serum E3, but not E1 or E2, the latter being more concerning in the context of breast cancer [181,182]. These findings indicate that, unlike the standard 0.5 mg E3 dose, administration of 0.03 mg E3 with *L. acidophilus* did not cause significant systemic absorption or clinically relevant elevations in estrogen levels [182]. In this context, the probiotic *L. acidophilus* served as an adjuvant, reducing the risks associated with higher doses of E3 while concurrently demonstrating efficacy in alleviating urogenital atrophy.

Altogether, these findings highlight that strain-specific probiotics, particularly lactic acid bacteria, can support estrogen balance, lipid metabolism, bone integrity, and cognitive and urogenital health. Nonetheless, large-scale clinical trials are required to confirm their efficacy and safety in women’s menopausal health.

#### 6.2.2. Prebiotics

A prebiotic is defined as “a substrate that is selectively utilized by host microorganisms conferring a health benefit”, and often, prebiotics are non-digestible soluble fibers serving as ‘food’ for beneficial microbes in the host gut. In general, prebiotics are known to modulate the gut microbiome to improve host health, including improving calcium absorption, regulating blood sugar and enhancing colonic SCFA production [183], which lowers colonic pH and improves calcium solubility and transporter expression [184].

In the context of postmenopausal women, fructo-oligosaccharide (FOS) or inulin-type fructan supplementation was associated with increased plasma isoflavone concentrations, enhancing the bioavailability of dietary phytoestrogens that may compensate for declining endogenous estrogen [185]. FOS also improved calcium absorption, therefore supporting bone health under estrogen-deficient conditions [186]. In addition, prebiotic consumption containing inulin and lactulose was reported to suppress bone resorption markers and increase the abundance of *Bifidobacterium* in young Japanese female athletes in a pilot study, conferring gut and bone health benefits [187]. Similarly, galacto-oligosaccharide (GOS) was reported to improve calcium bioavailability, mitigating bone loss and supporting skeletal health in postmenopausal women with reduced estrogen [188].

Lactulose is a prebiotic heat-treated lactose disaccharide that suppresses β-glucuronidase activity in the gut, thus influencing enterohepatic recirculation of estrogens [189]. In menopausal women, lactulose improved calcium uptake, a critical factor in counteracting estrogen-deficiency-related osteoporosis, and may reduce the reactivation of estrogen metabolites and lower the risk of estrogen-driven cancers [190]. Lactulose-mediated suppression of microbial β-glucuronidase activity may appear contradictory given the enzyme’s role in deconjugating estrogens for enterohepatic circulation. However, this effect is context dependent, as in hyperestrogenic conditions excessive β-glucuronidase activity can promote the reactivation of carcinogenic estrogen metabolites, whereas its suppression helps reduce estrogen-related oncogenic risks [191,192]. In postmenopausal women, where circulating estrogen levels are already low, the primary benefits of lactulose relate to improved calcium absorption, modulation of gut microbiota composition beyond estrogen recycling and reduced intestinal inflammation, therefore supporting bone and metabolic health.

In several preclinical models, prebiotic mixtures, including a mixture of FOS and GOS [193] and inulin and lactulose [187], showed promising results in promoting calcium absorption and for management of menopausal-related osteoporosis. Collectively, the literature supports prebiotic supplementation as an effective strategy to promote skeletal integrity and potentially reduce fracture risk for women’s health, with most evidence to date emphasizing its role in improving mineral bioavailability. Emerging findings as described above also point to broader gut microbial contributions within a complex gut–bone signaling network, but these interactions remain poorly elucidated. Taken together, the evidence indicates that prebiotics confer benefits to women across different life stages and hold promise for both preventive and therapeutic applications.

### 6.3. Diet, Phytoestrogens and Traditional Herbal Remedies

Phytoestrogens, plant-based compounds that are structurally similar to estrogen, have been studied for their potential therapeutic roles in women’s health. Phytoestrogens, such as isoflavones (from soybean) and lignans (from flaxseed), exert mild estrogenic or anti-estrogenic effects depending on endogenous hormone levels [194].

Lignan-rich flaxseed increases the 2-hydroxyestrone (2-OHE1)/16α-hydroxyestrone (16α-OHE1) ratio, favoring anti-estrogenic metabolism and potentially reducing hormone-dependent cancers [195,196]. 2-OHE1 is regarded as anti-estrogenic, whereas 16α-OHE1 is considered pro-carcinogenic; a lower ratio is posited to reflect a predominance of more biologically active 16α-OHE1, associated with an increased risk of cancer [197]. Isoflavones, like daidzein and genistein, provide vasomotor relief and osteoprotective and urogenital protective effects [194,198], though their efficacy is typically modest compared to HRT [199,200].

It is important to highlight that phytoestrogen bioactivity depends on gut microbial conversion. Certain bacteria convert daidzein, a soy isoflavone, into equol, a metabolite with stronger estrogenic potential [48,201,202]. Only “equol producers”, i.e., women whose gut microbiome contains bacteria that can produce equol, experience significant reductions in hot-flash frequency and severity, as demonstrated in clinical trials [203,204]. Phytoestrogen also influences microbial balance by enriching beneficial taxa such as *Faecalibacterium prausnitzii* and *Enterococcus* [202]. Beyond phytoestrogens, increased dietary fiber intake has been shown to support gut stability and symptom relief [205]. A 12-week low-fat, plant-based vegan diet improved hot-flash severity [206], while high fiber and lower glycemic index (GI) diets correlated with reduced menopausal symptom burden [207].

Traditional Chinese medicinal herbs like Dan Shen (*Salvia officinalis*) and Kudzu root (*Pueraria lobata*) exhibit both microbiome-modulating and estrogenic properties. Dan Shen promotes beneficial gut bacteria and alleviates vasomotor symptoms, with clinical studies reporting an approximately 39% reduction in Menopause Rating Scale scores and improvement in E2 levels [208,209,210]. Kudzu root, rich in puerarin, daidzein and genistein, improves gut microbial diversity and reduces oxidative stress, showing significant symptom relief in human trials [211,212,213,214,215]. Ashwagandha (*Withania somnifera*) also demonstrates potential for mood and urogenital symptom improvement via anti-inflammatory pathways (cyclooxygenase-2 (COX-2) and interleukin-8 (IL-8) inhibition) [216,217,218]. In addition, a combination of *Ashokarishta*, *Ashwagandha Churna* and *Praval Pishti* showed comparable results to HRT in alleviating menopausal-related symptoms, highlighting its potential as a viable alternative [216].

Despite these promising findings, further research is needed to elucidate the interrelated roles of dietary interventions, gut microbial dynamics, and menopausal symptom trajectories. Longitudinal studies that concurrently monitor dietary intake, individual differences in gut microbiota composition, and symptom progression will be crucial to clarify these complex interactions and inform the development of individualized, non-hormonal therapeutic strategies for menopause symptom management.

### 6.4. Fecal Microbiota Transplantation (FMT)

Fecal microbiota transplantation (FMT) is an emerging treatment approach to several diseases that feature a dysbiotic gut microbiome, specifically by restoring the dysbiotic gut microbiome to a healthy community state [219]. FMT has over 90% success rates in treating *Clostridium difficile* infections [220] and has been applied to various other gastrointestinal indications with varying success rates. Preclinical models suggest the potential of FMT for menopause-related disorders. Ma et al. did a 12- and 24-week course of FMT from young rats to aged rats with senile osteoporosis and found that the bone loss was significantly reduced through modification of the gut microbiota composition and improvement of intestinal barrier function [221]. Zhang et al. did an 8-week course of FMT from healthy mice to mice with ovariectomy-induced osteoporosis and similarly found modifications in the gut microbiome composition and the gut barrier permeability [222]. In addition, SCFA levels were elevated and release of pro-osteoclastogenic cytokines was suppressed, which collectively prevented ovariectomy-induced bone loss in these mice [222]. Huang et al. did an 8-week course of FMT from healthy female mice to ovariectomized mice and found that vaginal atrophy was alleviated, possibly through stimulating cell proliferation in the epithelial layer [223]. While these findings are encouraging, multiple clinical barriers contribute to the lack of robust human studies. These include donor–recipient matching and route of administration (colonoscopy vs. enema vs. oral capsules or granules vs. nasogastric tubes), which can affect patient acceptance, safety and effectiveness [219]. While rare, safety concerns with the use of FMT include unintended introduction of opportunistic infections [224]. As such, rigorous clinical evaluation is needed to establish FMT as a viable therapeutic strategy for menopause-associated conditions.

Across therapeutic modalities, interventions that restore microbial diversity, whether through HRT, probiotics, prebiotics, dietary phytoestrogens, herbal remedies, or FMT, show potential to mitigate menopausal-related symptoms. Although non-hormonal options for perimenopause management are generally safer in the long term than systemic HRT [225], their long-term safety similarly depends on individualized dose, duration, and comorbidity considerations. Nonetheless, the convergence of hormonal balance and microbiome modulation suggests a promising avenue for individualized, non-hormonal strategies to enhance women’s health during perimenopause and beyond.

## 7. Conclusions and Future Directions

While the gut microbiome, including the estrobolome, has been associated with perimenopause and manifestation of associated symptoms, studies that have demonstrated a causal relationship remain few and nascent. To date, there is a lack of consistent microbiome features across populations based on symptom severity. This might be attributed to differences in geography, age and subjectivity in self-reported measures. Furthermore, both gut microbiome profiles [226] and perimenopause symptom prevalence vary across ethnicity and geography [35], which may further confound the validity of associations observed in Western populations. Hence, it is critical to perform longitudinal studies of women across different perimenopause stages and symptom severity, particularly in non-Western geographies, to elucidate how host factors explain the large inter-individual variation in symptoms, hormone levels and gut microbiome profiles. With such insights, we would be able to design better approaches to identifying women who will benefit from microbiome-mediated adjuvants.

In addition to the identification of gut microbial biomarkers and mediators of perimenopausal symptom severity and comorbidities, elucidating the impact of diet and synbiotics (prebiotic, probiotic and postbiotic) on the gut microbiome and symptom manifestation will help design better microbiome-mediated dietary approaches as adjuvants or alternatives to HRT in managing perimenopause symptoms. Taken together, an improved understanding of the localized diet–microbiome interactions and their effects on perimenopausal symptoms offers opportunities to develop accessible solutions that leverage actionable, localized insights to improve women’s health throughout the perimenopause window.

## Figures and Tables

**Table 1:** Summary of studies of menopause and gut microbiome in women. The inclusion criteria for these studies were as follows: comparison of gut microbiome and estrogen levels after controlling for age between (1) pre- and postmenopausal women; (2) postmenopausal women with and without menopausal symptoms; (3) perimenopausal women with high or low levels of FSH. fc = fold-change.

Source	Country	Study Size	Control for Age	Method	Gut Microbiome Results	Estrogen Results
Zhao et al. (2019) [115]	China	*n* = 24 premenopausal women and *n* = 24 postmenopausal women	Matching	Shotgun metagenomic sequencing	Lower gene counts (fc = 0.737) and Shannon diversity index (fc = 0.948) in postmenopausal women Difference in beta diversity Depletion of *Firmicutes* (fc = 0.551) and *Roseburia* (fc = 0.707) in postmenopausal women Enrichment of *Bacteroidetes* (fc = 1.43) and *Tolumonas* (fc = 1.18) in postmenopausal women	N/A
Mayneris-Perxachs et al. (2020) [116]	Spain	*n* = 44 premenopausal women and *n* = 45 postmenopausal women	Multivariable adjustment	Shotgun metagenomic sequencing	No difference in overall alpha and beta diversity 90 differentially abundant taxa between pre- and postmenopausal women, such as depletion in *Ruminococcus* sp. CAG:379, *Clostridium neonatale*, *Bifidobacterium angulatum*, *Blautia* sp. CAG:257 (fc = 0.0186, 0.0266, 0.0486, 0.0409) and enrichment in *Bacteroides* sp. CAG:661, *Haemophilus* sp. HMSC71H05, *Prevotella* sp. P6B1, *Dorea* sp. CAG:317 (fc = 18.2, 15.1, 8.13, 9.30)	N/A
Peters et al. (2022) [98]	U.S.A.	*n* = 295 premenopausal women and *n* = 1027 postmenopausal women	Multivariable adjustment	Shotgun metagenomic sequencing	Lower Shannon diversity index in postmenopausal women (β = −0.079) Difference in beta diversity Depletion of *Escherichia coli-Shigella* spp., *Akkermansia muciniphila*, *Oscillibacter* sp. strain *KLE1745*, *Escherichia coli*, *Parabacteroides johnsonii*, *Clostridium lactatifermentans* and *Veillonella seminalis* (β = −1.101, −0.644, −0.335, −0.583, −0.345, −0.342, −0.534) Enrichment of *Sutterella wadsworthensis*, *Bacteroides* sp. strain *Ga6A1* and *Prevotella marshii* (β = 0.765, 0.543, 0.919)	N/A
Liu et al. (2022) [113]	China	*n* = 24 healthy postmenopausal and *n* = 77 postmenopausal women with menopausal symptoms	None (but age similar between groups)	16S rRNA gene sequencing	No difference in alpha and beta diversity 14 differentially abundant species between healthy and symptomatic postmenopausal women, such as depletion in *Aggregatibacter segnis*, *Acinetobacter guillouiae, Bifidobacterium animalis*, *Bacteroides coprophilus*, *Clostridium celatum*, *Ruminococcus albus* (LDA log_10_ score between −2 and −4), and enrichment in *Bifidobacterium adolescentis*, *Bifidobacterium longum*, *Bacteroides ovatus*, *Eubacterium biforme* (LDA log_10_ score between 2.1 and 4.3)	Higher estrogen, higher abundance of *Aggregatibacter segnis*, *Bifidobacterium animalis* and *Acinetobacter guillouiae* (*r* = 0.253, *p* = 0.018)
Xie et al. (2024) [114]	China	*n* = 16 perimenopausal women with low FSH level (<40 IU/L) and *n* = 28 perimenopausal women with high FSH level (>40 IU/L)	None (but age similar between groups)	16S rRNA gene sequencing	No difference in alpha and beta diversity Depletion of *Faecalibacterium*, *Subdolibranulum*, *Agathobacter*, unclassified *Lachnospiraceae*, *Roseburia* (fc = 0.676, 0.819, 0.433, 0.672, 0.647) in postmenopausal women with high FSH level at genus level Enrichment of *Bacteroides*, *Escherichia-Shigella*, *Bifidobacterium*, *Blautia* (fc = 1.24, 1.20, 1.49, 1.45) in postmenopausal women with high FSH level at genus level	Higher E2, higher abundance of *Monologues*, *Facalibaterium*, *Dialister*, and *unclassified Lachnospiraceae*

## Data Availability

No new data were created or analyzed in this review article. Data sharing is not applicable to this article.

## Abbreviations

The following abbreviations are used in this manuscript:

2-OHE1	2-hydroxyestrone
16α-OHE1	16α-hydroxyestrone
BMI	Body mass index
BV	Bacterial vaginosis
CFU	Colony forming units
COX-2	Cyclooxygenase-2
ER	Estrogen receptor
FAO	Food and Agriculture Organization
FC	Fold-change
FMT	Fecal microbiota transplantation
FOS	Fructo-oligosaccharide
FSH	Follicle stimulating hormone
GABA	γ-aminobutryic acid
GI	Glycemic index
GLP-1	Glucagon-like pepide-1
GOS	Galacto-oligosaccharide
GSM	Genitourinary syndrome of menopause
HDL	High-density lipoprotein
HRT	Hormone replacement therapy
IL-8	Interleukin-8
KI	Kupperman index
LDL	Low-density lipoprotein
LOOP	Luteal out-of-phase
MPS	Menopausal syndrome
PFC	Perfluorocarbon
PCOS	Polycystic ovary syndrome
PM_2.5_	Particulate matter 2.5
SCFA	Short-chain fatty acids
T2D	Type 2 diabetes
VMS	Vasomotor symptom
WHO	World Health Organization
